# Solar radiation drives methane emissions from the shoots of Scots pine

**DOI:** 10.1111/nph.18120

**Published:** 2022-04-12

**Authors:** Salla A. M. Tenhovirta, Lukas Kohl, Markku Koskinen, Marjo Patama, Anna Lintunen, Alessandro Zanetti, Rauna Lilja, Mari Pihlatie

**Affiliations:** ^1^ Department of Agricultural Sciences Environmental Soil Science University of Helsinki PO Box 56 Helsinki 00014 Finland; ^2^ Institute for Atmospheric and Earth System Research (INAR)/Forest Sciences University of Helsinki Helsinki 00560 Finland; ^3^ Department of Forest Sciences University of Helsinki PO Box 27 Helsinki 00014 Finland; ^4^ Department of Agricultural Sciences Viikki Plant Science Centre (ViPS) University of Helsinki Helsinki 00014 Finland

**Keywords:** aerobic methane production, boreal forests, evergreen trees, methane (CH_4_), plant‐mediated emissions

## Abstract

- Plants are recognized as sources of aerobically produced methane (CH_4_), but the seasonality, environmental drivers and significance of CH_4_ emissions from the canopies of evergreen boreal trees remain poorly understood.
- We measured the CH_4_ fluxes from the shoots of *Pinus sylvestris* (Scots pine) and *Picea abies* (Norway spruce) saplings in a static, non‐steady‐state chamber setup to investigate if the shoots of boreal conifers are a source of CH_4_ during spring.
- We found that the shoots of Scots pine emitted CH_4_ and these emissions correlated with the photosynthetically active radiation. For Norway spruce, the evidence for CH_4_ emissions from the shoots was inconclusive.
- Our study shows that the canopies of evergreen boreal trees are a potential source of CH_4_ in the spring and that these emissions are driven by a temperature‐by‐light interaction effect of solar radiation either directly or indirectly through its effects on tree physiological processes.

## Introduction

Vegetation has recently been recognized as an important component of the global cycle of methane (CH_4_), a powerful greenhouse gas (Carmichael *et al*., 2014). Plants, both woody and herbaceous, can contribute to the CH_4_ exchange of different ecosystems via various pathways: soil‐derived CH_4_ can be exported through stems (Pangala *et al*., 2013, 2015; Barba *et al*., 2019), CH_4_ may be produced aerobically in foliage (Keppler *et al*., 2006; Brüggemann *et al*., 2009; Qaderi & Reid, 2009, 2011; Wang *et al*., 2009; Fraser *et al*., 2015; Martel & Qaderi, 2017, 2019), and it may be produced by microbial methanogenesis within core wood and potentially also other plant tissues (Yip *et al*., 2019; Putkinen *et al*., 2021). These fluxes, however, have not been incorporated into global CH_4_ budgets as fundamental questions about the mechanisms, drivers and quantities of plant CH_4_ emissions remain unaddressed (Saunois *et al*., 2020).

Boreal forests cover *c*. 30% of the global forest area (Brandt *et al*., 2013), presenting an unignorable potential source of CH_4_. Recent research shows that trees in temperate and boreal forests emit CH_4_ in natural conditions (Covey *et al*., 2012; Machacova *et al*., 2016; Pitz & Megonigal, 2017; Covey & Megonigal, 2019) and can thus decrease the overall sink strength of upland forests, which is sustained by the oxidative consumption of CH_4_ in the soil (Ito & Inatomi, 2012). Most of the research on CH_4_ emissions from trees, however, has focused on emissions of soil‐derived CH_4_ from tree stems in tropical or temperate wetland forests (Terazawa *et al*., 2007; Pangala *et al*., 2013, 2015; Jeffrey *et al*., 2020). By contrast, relatively little is known about CH_4_ emissions from the trees of the evergreen boreal forests, especially with respect to the potential aerobic *in situ* production and environmental drivers of CH_4_ emission in the canopies. This lack of knowledge prevents us from constructing models for upscaling canopy emissions from regional scales to the global scale.

Emissions of CH_4_ from forest canopies likely derive primarily from aerobic production in the foliage, but the biochemical origin and the source processes involved remain poorly understood. Laboratory studies with purified plant compounds and plant parts have demonstrated that compounds like pectin (Keppler *et al*., 2008; McLeod *et al*., 2008; Bruhn *et al*., 2009), lignin, cellulose (Vigano *et al*., 2008) and methionine (Althoff *et al*., 2014; Lenhart *et al*., 2015) can serve as precursors for CH_4_ emissions in aerobic conditions, which are induced by various environmental drivers. Current evidence indicates that the unifying factor for aerobic CH_4_ emissions from plants is the increased activity of reactive oxygen species (ROS) (McLeod *et al*., 2008; Messenger *et al*., 2009a,b), which is induced by environmental plant stressors such as ultraviolet (UV) radiation and heat (Bruhn *et al*., 2009; Qaderi & Reid, 2009; Fraser *et al*., 2015), and wounding of plant tissues (Wang *et al*., 2009). Despite the numerous studies focusing on potential precursors and drivers in laboratory settings, research on aerobic CH_4_ emission from intact plants in ambient outdoor conditions remains rare.

Fluxes of CH_4_ from the canopies of evergreen trees have only been reported in a handful of field studies. These results vary from small emissions early in the summer from Scots pine (Machacova *et al*., 2016) to both small emissions and apparent uptake by Norway spruce in the summer (Putkinen *et al*., 2021), and uptake in low light conditions late in the fall (Sundqvist *et al*., 2012). It remains unknown how the high seasonal variations typical to the boreal climate affect the canopy CH_4_ fluxes. Adaptation to the seasonal changes involves drastic alterations to the metabolic processes of evergreen trees which, in the spring in particular, affect their gas exchange and stress tolerance (Hari *et al*., 2017). For example, the emissions of volatile organic compounds (VOC) and methanol, a compound structurally similar to CH_4_, from vegetative buds and young shoots of Scots pine trees have been shown to peak in the spring and early in the summer (Aalto *et al*., 2014). Moreover, boreal conifers are exposed to photochemical stress under cold temperatures early in the spring, before their photosynthetic machinery has adapted to the increasing light levels (Ottander *et al*., 1995; Vogg *et al*., 1998; Yang *et al*., 2020).

In this study, we aim to provide missing information about the canopy CH_4_ exchange of conifers in the spring. The overall objective of this study was to investigate whether the shoots of boreal conifer trees are a source of CH_4_ in the spring, and to examine whether solar radiation and ambient air temperature affect the CH_4_ exchange. We hypothesize that Scots pine and Norway spruce shoots act as net CH_4_ sources in the spring, that CH_4_ emissions increase under higher light and warmer temperature conditions within the ambient range, and that CH_4_ emissions increase over the spring with the increasing physiological activity of the trees.

To test our hypotheses, we measured the CH_4_ exchange from the shoots of Scots pine and Norway spruce saplings early in the growing season in 2019 and 2020. Simultaneous with the CH_4_ flux measurements, we measured the environmental variables solar radiation and air temperature, and followed tree physiological activity during the breaking of winter dormancy.

## Materials and Methods

### Experimental design

The study was conducted in ambient conditions with potted 2–3‐yr‐old nursery saplings of *Pinus sylvestris* L. (Scots pine) and *Picea abies* (L.) Karst. (Norway spruce). The measurement site was located in Helsinki, Finland, in the courtyard of the Viikki Plant Growth Facility (lat. 60°13′40′′N, long. 25°01′05′′E). The measurements were made before and during the early growing season and repeated in two springs: 1 April–8 May in 2019 and 6 March–9 June in 2020. In 2019, we measured over a shorter period of time to confirm the CH_4_ emissions, and in 2020, we measured from late winter to early summer to cover the period from the spring recovery to the beginning of the annual growth.

The balled and burlapped saplings were acquired in the autumn from commercial nurseries (Huutokoski Ltd (in 2019) and Harviala Ltd (in 2020)) and planted into 15–20 l plastic pots with a mixture of peat and humus, 6 months before the experiment. The potted saplings were planted, with their pots, in a sand bed outdoors between the glasshouses to overwinter. To avoid the formation of anaerobic conditions in the soil – in order to prevent potential anaerobic microbial methanogenesis occurring during the measurements – we used pots with drainage holes and irrigated the saplings only moderately; the saplings were kept in ambient outdoor conditions throughout the winter and during the experiment, except for the use of irrigation after longer dry periods, twice in spring 2019 and three times in spring 2020.

In both years, the gas exchange of four Scots pine and three Norway spruce shoots were repeatedly measured, each year with new saplings. The measurements were made nine times per week (three measurements each on 3 d) in 2019 and four times per week (two measurements each on 2 d) in 2020. An empty chamber was measured as a control at the beginning of each measurement round. Overall, there were 181 Scots pine, 53 Norway spruce and 55 empty chamber measurements made in 2019, and 205 Scots pine, 89 Norway spruce and 46 empty chamber measurements made in 2020. A brief summary of the CH_4_ fluxes from Norway spruce shoots measured in 2020 was presented previously by Putkinen *et al*. (2021); however, so far, no thorough analysis of this dataset has been reported. Our data from 2019 or from the Scots pine measurements of 2020 have not been presented before.

### CH_4_ and CO_2_ flux measurements

To quantify the greenhouse gas exchange of the shoots and the background emissions potentially produced by chamber materials, CH_4_ and CO_2_ fluxes were measured using a manual chamber system, including transparent cylindrical shoot chambers (described in Machacova *et al*., 2016) and a portable greenhouse gas analyser (ABB Ultraportable Greenhouse Gas Analyzer; Los Gatos Research, San Jose, CA, USA). Each chamber (volume of 5.2 l) consisted of a permanently installed frame with a circular opaque bottom and top pieces made of polytetrafluoroethylene (PTFE) held together by four metal bars. The measured shoot and a fan to mix the headspace air were enclosed in the chamber; the chamber was then covered by a UV‐transparent fluorinated ethylene propylene‐foil (FEP) during each flux measurement. The opening for the stem in the bottom piece was sealed with adhesive putty (Blu Tack; Bostik SA, Colombes, France). The fluxes were measured in 7–10‐min long chamber closures, during which the headspace air of the chamber was circulated in a closed loop between the analyser and the chamber. To maintain the shoots in ambient conditions, the FEP‐foil was removed between each measurement.

### Meteorological variables

Meteorological variables were continuously measured at the weather station of the Viikki Plant Growth Facility. The weather station is positioned on the glasshouse roof, at a height of *c*. 7 m from the ground. The ambient air temperature (°C), global radiation (W m^−2^), and photosynthetically active radiation (PAR; µmol^−1^ m^−2^ s^−1^) were measured with an outside temperature sensor, a CM3P thermopile radiation sensor (both manufactured by Priva Agro, De Lier, the Netherlands) and an LI‐190 PAR sensor (Li‐Cor, Lincoln, NE, USA). The meteorological variables were averaged over 15 min in 2019 and 5 min in 2020. The total daily precipitation values for 2020 were obtained from the Kumpula measurement station of the Finnish Meteorological Institute (FMI), which is located *c*. 4 km from the measurement site.

### Spring recovery of the saplings

To follow the spring recovery of the saplings in 2020, we monitored them for the seasonal increase in photosynthetic activity and changes in water status (Leinonen & Hänninen, 2002; Lintunen *et al*., 2018). The measurements were conducted on the same days as the gas exchange measurements, but in different individuals (four Scots pine and four Norway spruce saplings) to minimize any potential disturbance of the gas exchange measurements.

The photosynthetic potential of the saplings was defined by measuring the Chl*a* fluorescence to analyse the maximum efficiency of photosystem II (PSII, *F*_v_/*F*_m_). The measured needles were first enclosed in lightproof clips for an hour to stop all photosynthetic reactions. Chlorophyll *a* fluorescence was then measured with the Field Fluorescence Monitoring System (FMS2+; Hansatech, King's Lynn, UK), which applies a combination of low intensity light, to measure the minimum fluorescence (*F*_0_), and brief saturating flashes (< 1 s), to measure the maximum fluorescence (*F*_m_), from which *F*_v_/*F*_m_ is calculated as follows:

(Eqn 1) $$F_{v}/F_{m}=\left(F_{m}‐F_{0}\right)/F_{m}$$

Low values of *F*_v_/*F*_m_ indicate that the light reactions are downregulated after the winter, whereas high values indicate that the photosynthetic apparatus is active (Linkosalo *et al*., 2014).

To assess the water status of the saplings, the needle water potential (Ψ) was measured at *c*. 09:00 h on the measurement days. A pair of needles was cut from each sapling, sealed into a plastic bag to avoid evaporation and immediately taken to the laboratory to be measured. A scalpel was used to apply a fresh cut to the needle sample before measuring the needle Ψ using a pressure chamber (I505D‐EXP; PMS Instrument Co., Albany, OR, USA).

### Shoot sample processing and growth estimation

To record the growth of the current‐year (*Y*_0_) shoot during the measurement campaign in 2020, the terminal buds developing into *Y*_0_ shoots of the Scots pine saplings were photographed and their length was measured weekly. To obtain the biomasses of the foliage in the chambers, the shoots were collected once the campaign was over. The 1‐yr‐old (*Y*_1_) and *Y*_0_ needles were separated from the branches, oven‐dried at 65°C for 72 h and weighed.

To calculate the gas fluxes per unit needle biomass, the *Y*_0_ needle masses, which increased throughout the measurement campaigns, were estimated for each measuring day using the relative growth of the *Y*_0_ shoots (i.e. shoot length on measurement day/shoot length at the end of the campaign). For this, we assumed that needle biomass increases proportionally with the elongation of the *Y*_0_ shoot. For the Scots pine saplings of 2020, the relative growth was based on the measured shoot elongation growth. For the Norway spruce saplings of both years and the Scots pine saplings of 2019, the *Y*_0_ relative growth was estimated using a model (Schiestl‐Aalto *et al*., 2013) that describes the elongation growth of Scots pine shoots with respect to cumulative growing degree days above 5°C. To validate the suitability of the model for predicting the growth of our saplings, the relative growth, based on the measured *Y*_0_ shoots in 2020, was compared to the relative growth obtained from the model in spring 2020.

### Gas flux calculations

Each measurement closure was examined graphically, with gas concentrations plotted as a function of time. This was done to adjust the start and end times of the flux calculation in order to omit the values corresponding to instability in the measurement loop caused by mixing of the headspace gases at the beginning of the closure, leakage of the chambers, and inaccuracies in the recorded start and end times (Supporting Information Fig. S1). Closures with poor data quality were discarded (8.6% of all closures in 2019 and 20.8% in 2020).

During the measurements, we noticed that the greenhouse gas analyser’s automatic correction for H_2_O spectral interferences did not work accurately: increases in H_2_O concentrations led to apparent decreases in the measured CH_4_ concentrations. To correct for this source of error in the CH_4_ measurements, we conducted a laboratory experiment to empirically determine a correction factor *f* for the H_2_O interference: A 0.8 l empty chamber equipped with an injection septum was flushed with dry air and then connected in a closed loop with the analyser. Next, *c*. 1 ml of water was injected to the chamber. As the measurement loop air became saturated with water vapour, the humidity in the analyser’s measurement chamber increased rapidly, while the (actual) dry CH_4_ mixing ratios remained unchanged; *f* was determined as the regression slope between the measured dry CH_4_ mixing ratio and the mixing ratio of H_2_O. The experiment was replicated six times, resulting in a value *f* (−9.122 × 10^–7^ ppm CH_4_ ppm^−1^ H_2_O, 95% confidence interval 0.452 × 10^–7^ ppm CH_4_ ppm^−1^ H_2_O), which was used to correct the measured CH_4_ concentration values:

(Eqn 2) $${\left[{\text{CH}}_{4}\right]}_{\text{dry},\text{corr}}\left(t\right)={\left[{\text{CH}}_{4}\right]}_{\text{dry},\text{raw}}\left(t\right)‐\left[H_{2}O\right]\left(t\right)\cdot f$$

The flux rates of CH_4_ from shoots and the empty chamber were calculated using the least squares method, defining the best linear fit for the concentration change over the closure time (d*C*/d*t*). The hourly flux rates from the shoots were then calculated using d*C*/d*t* and normalized to the dry weight of the needles of each chamber‐enclosed shoot:

(Eqn 3) $$F=\frac{dC}{dt}\cdot \frac{M\cdot V}{m_{\text{needles}}}\cdot \frac{p}{R\cdot T}\cdot 3600$$

*F*, CH_4_ flux (ng g^−1^ (needle dry weight) h^−1^); *M*, molar mass of CH_4_ (1.604 × 10^10^ ng mol^−1^); *m*_needles_, needle mass (g dry weight of *Y*_0_ + *Y*_1_ needles taking into account to the growth over the campaign); *p*, pressure (assumed 101 325 Pa); *R*, gas constant (8.31446 J mol^−1^ K^−1^); *T* (K), sample temperature as it enters the analyser; *V*, shoot chamber volume including the loop volume (5.2 × 10^–3^ m^3^).

The CO_2_ flux was estimated using an exponential function fitted to the concentration over time (*t*):

(Eqn 4) $$C\left(t\right)=C_{\lim}+\left(C_{0}‐C_{\lim}\right)\cdot e^{‐k\cdot t}$$

*C*_0_, initial concentration; *C*_lim_, the asymptote; *e*, Euler’s number (2.71828); *k*, rate constant. The initial d*C*/d*t* was calculated as the initial slope of the following function:

(Eqn 5) $$\frac{dC}{dt}=‐k\cdot \left(C_{0}‐C_{\lim}\right)$$

The CO_2_ flux was calculated in a similar manner to the CH_4_ flux (Eqn 3), but in terms of mg g^−1^ (needle dry weight) h^−1^ and using the molar mass of CO_2_ (4.401 × 10^4^ mg mol^−1^).

To correct for the background CH_4_ emitted by the chamber materials (Table S1), the CH_4_ fluxes of the empty chambers were first tested for their sensitivity to environmental variables by performing linear analyses for CH_4_ fluxes as functions of global radiation and air temperature. For 2020, when such sensitivities were not detected and the mean of the empty chamber CH_4_ did not significantly change over the campaign, the mean empty chamber CH_4_ flux was subtracted from the shoot chamber CH_4_ fluxes. For 2019, when the linear model revealed a significant correlation between the empty chamber CH_4_ flux and global radiation (as well as PAR), this model was used to predict the subtracted chamber background flux for each shoot chamber closure based on the corresponding mean global radiation.

Finally, the method detection limit (MDL) for a single chamber closure was defined as three times the SD of the apparent CH_4_ flux measured for the empty chambers. Separate detection limits were calculated for 2019 (123.3 ng CH_4_ h^−1^) and 2020 (120.9 ng CH_4_ h^−1^). When scaled to the average shoot dry weight for each species and each year, the MDL ranged from 5.12 to 27.2 ng CH_4_ g^−1^ dry weight (DW) h^−1^ (Table 1). These detection limits decrease with $\sqrt{n}$ for repeated measurements, resulting in detection limits between 0.73 and 6.48 ng CH_4_ g^−1^ DW h^−1^ for the average number of measurements conducted over a 2‐wk period.

**Table 1:** Method detection limits, average dry weight (DW) of the shoots, range and median of the number of measurements per 2‐wk period, and detection limits for the measurements of these 2‐wk periods.

Measure	Unit	Year		Tree species
		2019	2020	
Detection limit per chamber^a^	ng CH_4_ h^−1^	120.9	123.3	–
Detection limit per measurement^b^	ng CH_4_ g^−1^ DW h^−1^	5.12	27.2	Scots pine
		21.9	25.9	Norway spruce
Average shoot DW	g	23.6	4.54	Scots pine
		5.53	4.76	Norway spruce
Median of measurements (*n*) per 2‐wk period		49	27	Scots pine
		17	16	Norway spruce
Range of measurements (*n*) per 2‐wk period		49–83	24–39	Scots pine
		16–20	5–20	Norway spruce
Detection limit per 2‐wk period^c^	ng CH_4_ g^−1^ DW h^−1^	0.73	5.23	Scots pine
		5.30	6.48	Norway spruce

^a^ Detection limit for the amount of CH_4_ released into the enclosure chamber during a single closure (not scaled to shoot size), defined as three times the SD of the emissions measured in the empty chamber.

^b^ Typical detection limit for CH_4_ emissions from a spruce or pine shoot, defined as the detection limit described in (^a^) divided by the average shoot dry weight for each year and species.

^c^ Typical detection limit for the average flux of repeated measurements per 2‐wk time period, defined as the detection limit described in (^b^) divided by the square root of the number of replicate measurements (median of the number of measurements per 2‐wk period).

### Statistical analysis

To assess the trends in CH_4_ fluxes over the two springs, the data were divided into chronological subgroups, each consisting of measurements from a 2‐wk period. The differences in the mean CH_4_ fluxes between these subgroups were tested with a linear mixed‐effects analysis using the individual tree as a random intercept, and multiple comparisons of means. Further, the differences between the CH_4_ emission of each shoot chamber and the empty chamber background emission were tested with an independent *t*‐test.

To test if the CH_4_ fluxes were influenced by environmental variables or the uptake rate of CO_2_ in each tree species, we performed a linear mixed‐effects analysis, where the model was used to express the correlation of CH_4_ fluxes as a function of CO_2_ fluxes, PAR, global radiation, and ambient air temperature. Further, the relationship between PAR and global radiation was tested with a linear model. As with the analysis of the subgroups, the nonindependence of the data points of the repeated measurements was solved by adding a random effect for individual trees. In addition, the covariance of PAR and the temperature was analysed with a linear regression model.

To distinguish between PAR and air temperature as the potential drivers of CH_4_ emissions, the data were divided into groups of low and high PAR values (PAR ≤ 500 and ≥ 500 µmol^−1^ m^−2^ s^−1^, respectively), after which the groups were analysed separately. To test for an interaction effect of air temperature and light on CH_4_ emissions, a factor variable was created which grouped the data into four bins based on temperature (every five degrees from 0°C to 20°C), after which Helmert contrasts were applied to the variable to enable testing between the subsequent levels. A linear mixed‐effects analysis was then performed using PAR, the temperature bins and the interaction between PAR and the bin variable as independent variables, and individual trees as random intercepts.

All data analysis was conducted using the R v.4.0.5 software environment (R Core Team, 2017), with the additional packages nlme v.3.1‐152 (Pinheiro *et al*., 2021), multcomp v.1.4‐17 (Hothorn *et al*., 2008) and lme4 v.1.1‐27.1 (Bates *et al*., 2015).

## Results

### Meteorological conditions

**Fig. 1:**
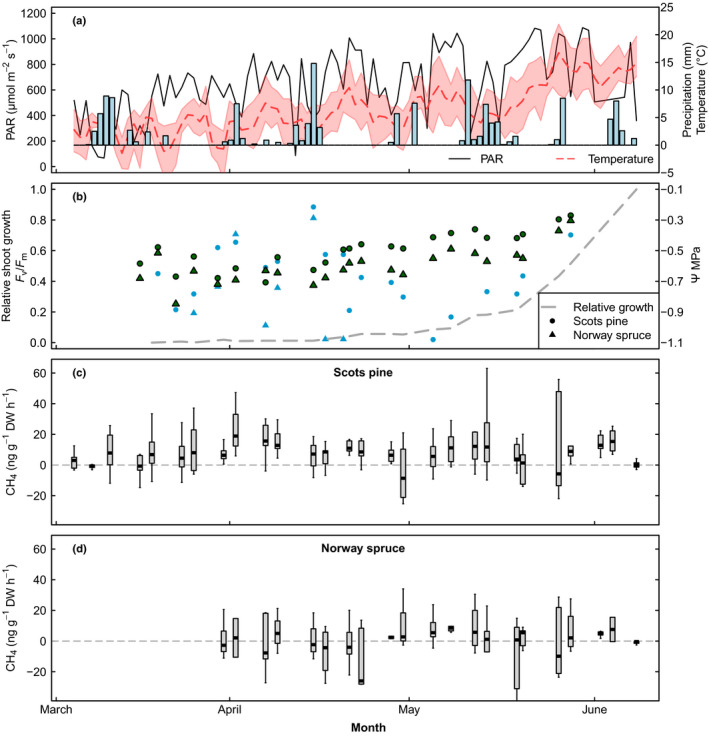
The weather conditions, physiological activity of the saplings, and daily CH_4_ fluxes from the shoots in the spring of 2020. (a) The daily average photosynthetically active radiation (PAR, µmol m^−2^ s^−1^), daily precipitation (mm) and daily average air temperature (°C, dashed line) and temperature range (red area) during the 2020 measurement campaign. (b) Spring recovery of the saplings (*F*_v_/*F*_m_, green symbols), the average relative growth of the Scots pine saplings (dashed line) and the mean needle water potentials of the saplings (Ψ, MPa, blue symbols). (c, d) The daily CH_4_ fluxes (ng g^−1^ dry weight h^−1^) of the Scots pine (c) and Norway spruce (d) measured in spring 2020. The horizontal lines in the boxes (b, d) indicate the median values, the boxed areas represent the inter‐quartile range, and the whiskers show the minimum and maximum values. The grey horizontal dashed lines indicate the zero‐line for CH_4_ fluxes.

In 2019, the start of spring, defined as the time when the average 24‐h temperature permanently exceeds 0°C, occurred on 13 March, and the thermal growing season started on 26 April (FMI). In spring 2020 (Fig. 1) the measurements followed a snowless and relatively mild winter. The spring started on the 1 March and the thermal growing season on 18 April in Helsinki. Photosynthetically active radiation and global radiation were collinear in both years (*R* ^2^ = 1.0, *P* < 0.001 in 2019 and *R* ^2^ = 0.98, *P* < 0.001 in 2020).

### Shoot growth and Chl*a* fluorescence

In the 2020 campaign when we followed the spring recovery of the trees, the first visible signs of the start of the new annual growth were noticed on 21 April. Based on the *F*_v_/*F*_m_ values, the spring recovery of the photosystem started simultaneously with the visible shoot growth and was complete at the end of May (Fig. 1b, *c*. 0.8 being a typical summer value for Scots pine and Norway spruce in Finland, Linkosalo *et al*., 2014). As for the water status of the saplings, the needle water potential increased after the winter dehydration, reaching a nearly saturated state in early April (Fig. 1b). From early April to late April, the water potential decreased down to −1.2 MPa possibly due to the fact that there was an increased transpiration demand but the cold soil was still limiting the water uptake of the saplings. From late April to early June, water potential started to increase again.

### Shoot methane fluxes

**Fig. 2:**
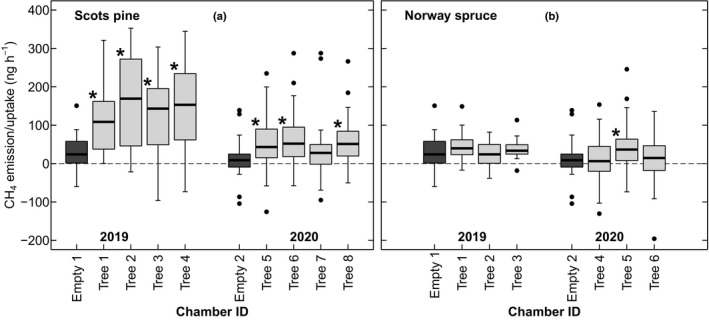
The apparent CH_4_ emission/uptake (i.e. not scaled to needle dry weight; ng h^−1^) in the empty measurement chambers (dark grey) with respect to chambers containing shoots (light grey) of Scots pine (a) or Norway spruce (b) in 2019 and 2020, before correction for the chamber background flux. The horizontal lines in the boxes represent median values, the boxed areas represent the inter‐quartile range, the whiskers show the minimum and maximum values, and the black dots are outlying values. The grey horizontal dashed line indicates the zero‐line for the apparent CH_4_ emission/uptake. Statistically significant differences between each shoot chamber and the empty chamber of the corresponding year are indicated by an asterisk (*, *P* < 0.05).

We observed CH_4_ emissions from the shoots of both Scots pine and Norway spruce in spring 2019 and spring 2020, of which the emissions of Scots pine could be verified statistically: CH_4_ emissions measured from chambers containing Scots pine shoots were significantly higher than fluxes measured from the empty chambers for all but one shoot chamber (Fig. 2a). The range of variation differed between the years and species: the flux rates of Scots pine ranged from 1.59 to 7.46 ng CH_4_ g^−1^ DW h^−1^ in 2019 and from −0.09 to 15.16 ng CH_4_ g^−1^ DW h^−1^ in 2020 (inter‐quartile range; IQR). The flux rates of Norway spruce ranged from −2.21 to 5.21 ng CH_4_ g^−1^ DW h^−1^ in 2019 and from −6.60 to 9.05 ng CH_4_ g^−1^ DW h^−1^ in 2020 (IQR). The CH_4_ flux of the empty chambers was 27.38 ± 5.44 ng h^−1^ (mean ± SE) in 2019 and 11.76 ± 6.06 in 2020. The empty chamber fluxes are presented relative to the shoot chamber fluxes (Fig. 2) before correcting for the background CH_4_ emitted by the chamber materials or scaling to shoot dry weight.

### Methane fluxes over the spring

**Fig. 3:**
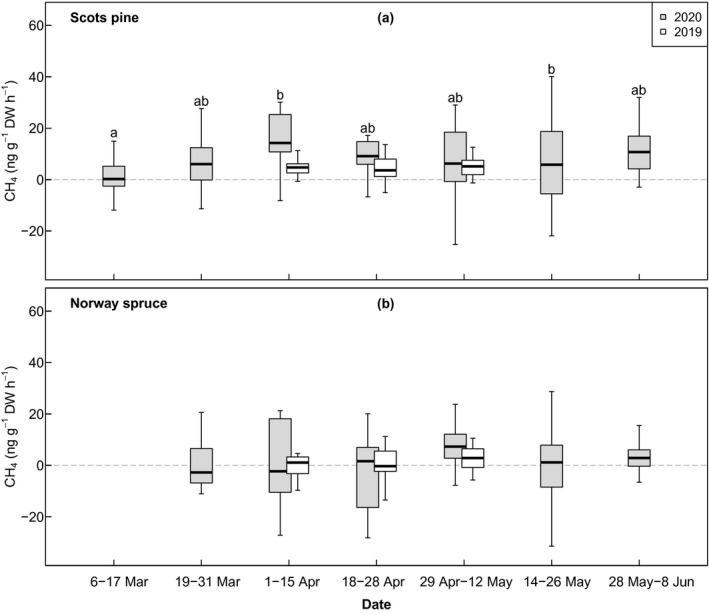
The variability of CH_4_ fluxes (ng g^−1^ dry weight h^−1^) of Scots pine (a) and Norway spruce (b) within temporal subgroups in spring 2020 (grey) and spring 2019 (white). The horizontal lines in the boxes indicate the median values, the boxed areas represent the inter‐quartile range, and the whiskers show the minimum and maximum values. The compact letter display (CLD) indicates the statistically significant differences between the temporal subgroups of Scots pine in 2020. The grey horizontal dashed line indicates the zero‐line for CH_4_ fluxes.

Over spring 2020, the CH_4_ emissions from Scots pine increased between March and mid‐April, after which the increase levelled off (Fig. 3a). When the fluxes were examined in groups based on pooled measurements from 2‐wk time intervals, the mean flux was lowest in early to mid‐March (Group 1, 1.75 ± 1.37 ng CH_4_ g^−1^ DW h^−1^, mean ± SE). In 2020, the highest mean positive fluxes were measured in early to mid‐April (Group 3, 16.1 ± 2.70 ng CH_4_ g^−1^ DW h^−1^), whereas in 2019 the fluxes remained almost identical in all three time groups throughout the campaign, from mid‐April to mid‐May (4.62 ± 0.46, 4.89 ± 0.67 and 4.93 ± 0.36 ng CH_4_ g^−1^ DW h^−1^, respectively). The mixed‐effects linear model and multiple comparison of means indicated a statistically significant difference in the CH_4_ fluxes in early to mid‐March 2020 (Group 1) in comparison to late‐April 2020 (Group 3, *P* = 0.001) and mid‐to‐late May 2020 (Group 6, *P* = 0.046). In 2019, no change in emission rates was observed during the campaign. As for the Norway spruce, no distinct changes in CH_4_ fluxes were confirmed in either year (Fig. 3b), although the measurements of late April to mid‐May 2020 suggest net CH_4_ emissions. The observed CH_4_ fluxes were lowest (0.90 ± 1.45 ng CH_4_ g^−1^ in 2019 and −4.34 ± 3.52 ng CH_4_ g^−1^ in 2020) in mid‐ to late April (Group 4).

### Methane and photosynthetically active radiation, solar radiation and air temperature

**Fig. 4:**
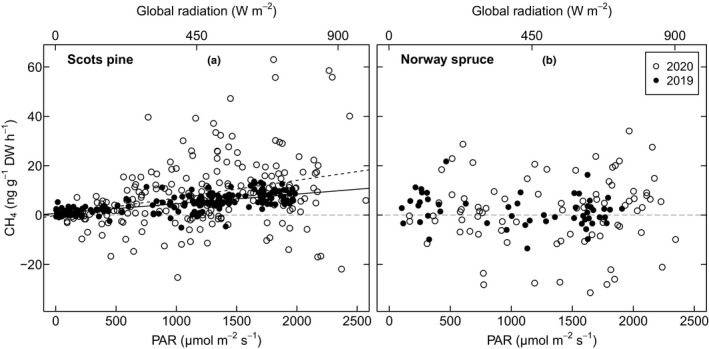
Mixed‐effects linear model fits showing the overall relationship of the CH_4_ fluxes (ng g^−1^ dry weight h^−1^) of Scots pine and Norway spruce with photosynthetically active radiation (PAR, µmol m^−2^ s^−1^) and global radiation (W m^−2^) in 2019 (black points and solid lines) and 2020 (white points, black dashed line). The statistics for the linear fits are for the fluxes as a function of PAR. For Scots pine (a) they are *P* < 0.001 for both years, *y* = 0.004*x* + 0.686 and *R* ^2^ = 0.45 for 2019 and *y* = 0.007*x* − 0.160 and *R* ^2^ = 0.09 for 2020. For Norway spruce (b) the model showed no statistical significance in either year. The grey horizontal dashed line indicates the zero‐line for CH_4_ fluxes.

The CH_4_ fluxes of Scots pine correlated positively with global radiation and PAR (Fig. 4a) in both 2019 and 2020 (*P* < 0.001). The relationship between PAR and CH_4_ fluxes was also examined between each of the time groups, but no statistically significant trend in this relationship was observed over the spring in either year.

**Fig. 5:**
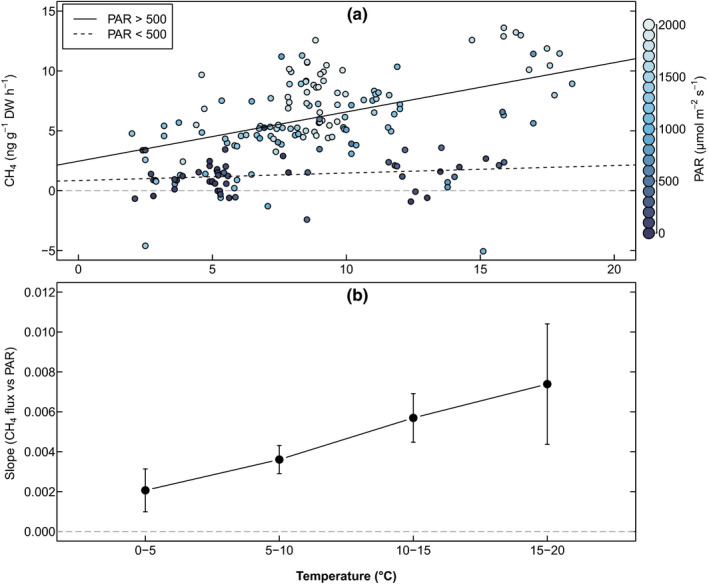
The interaction effect of photosynthetically active radiation (PAR) and temperature to CH_4_ emissions. (a) Linear model fits showing the relationship between ambient air temperature (°C) and CH_4_ emissions (ng g^−1^ dry weight h^−1^) for Scots pine in 2019, in groups of low (dashed line, *y* = 0.063*x* + 0.847, *P* = 0.19, *R* ^2^ = 0.032) and high (solid line, *y* = 0.412*x* + 2.46, *P* < 0.001, *R* ^2^ = 0.19) PAR (µmol m^−2^ s^−1^). (b) The slopes (±SE) of the mixed‐effects linear model fits for CH_4_ and PAR correlation, divided into four temperature bins. The grey horizontal lines indicate the zero‐line for CH_4_ emissions and slopes.

The CH_4_ fluxes of Scots pine also positively correlated with ambient air temperature in both 2019 (*R* ^2^ **= **0.19, *P* < 0.001) and 2020 (*R* ^2^ = 0.06, *P* = 0.009). To some extent, this correlation likely resulted from the co‐variance between air temperature and PAR (*R* ^2^ = 0.04, *P* < 0.01 in 2019 and *R* ^2^ = 0.23, *P* < 0.001 in 2020) as the regression residuals of fluxes with temperature were still correlated to PAR but not vice versa. In 2019, CH_4_ fluxes were only correlated with air temperature under high PAR conditions (≥ 500 µmol m^−2^ s^−1^, *R* ^2^ = 0.16, *P* < 0.001) (Fig. 5a). Furthermore, the slopes of the mixed‐effects linear model fits for CH_4_ and PAR correlation increased with air temperature between all of the temperature bins: using Helmert contrasts, all of the levels were significant (Fig. 5b).

For Norway spruce, the CH_4_ fluxes did not correlate with global radiation, PAR (Fig. 4b) or air temperature in either year.

### Methane emissions and photosynthesis

**Fig. 6:**
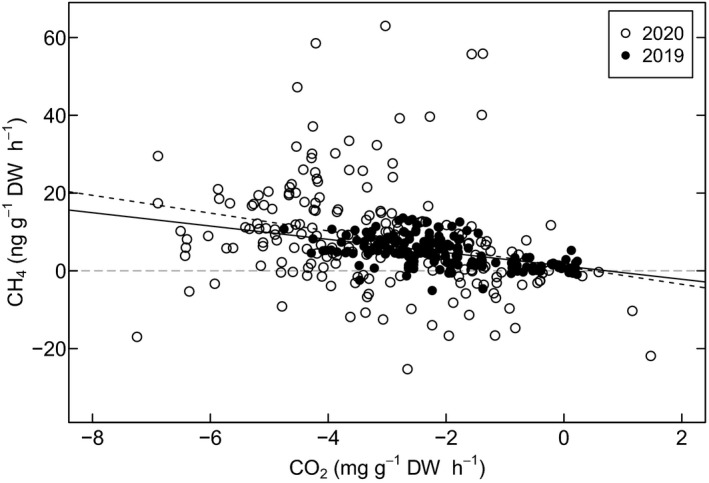
Mixed‐effects linear model fits of the correlation between CH_4_ fluxes (ng g^−1^ dry weight h^−1^) and CO_2_ emission (mg g^−1^ dry weight h^−1^) of Scots pine in 2019 (solid line) and 2020 (dashed line). The statistics of the linear fits are *y* = −1.712*x* + 1.254, *P* < 0.001, *R* ^2^ = 0.26 for 2019 and *y* = −2.290*x* + 1.111, *P* < 0.001, *R* ^2^ = 0.08 for 2020. The grey horizontal line indicates the zero‐line for CH_4_ emissions.

The CH_4_ emissions from the Scots pine correlated positively with CO_2_ uptake in both 2019 and 2020 (*R* ^2^ = 0.38 and 0.08 respectively, *P* < 0.0001) (Fig. 6). For Norway spruce, no correlation between CH_4_ fluxes and CO_2_ was detected in either year.

## Discussion

### The shoots of Scots pine are a source of CH_4_

In this study, we show that the shoots of Scots pine are a source of CH_4_ emissions. Our greenhouse gas measurements, conducted from the shoots of young saplings in a garden setup, showed CH_4_ emissions *c*. 20–30 times higher in sunlight (PAR ≥ 500 µmol m^−2^ s^−1^) and of the same order of magnitude in cloudy conditions (PAR ≤ 500 µmol m^−2^ s^−1^) as those observed from the shoots of mature Scots pine trees measured during overcast weather (Machacova *et al*., 2016) (0.225 ng CH_4_ g^−1^ DW h^−1^, assuming a specific leaf area of 45 cm^2^ g^−1^ DW (Xiao *et al*., 2006)). Our low‐PAR CH_4_ emissions were also similar in magnitude to those measured from poplar cultures under low‐light conditions (Brüggemann *et al*., 2009). We show CH_4_ emissions *c*. 2–3 times smaller than the lowest CH_4_ fluxes reported by Keppler *et al*. (2006), and of the same order of magnitude as those measured from plant biomass in laboratory experiments by Martel & Qaderi (2017).

Our results, together with those of Machacova *et al*. (2016), indicate that boreal conifer shoots have the potential for aerobic formation of CH_4_, a phenomenon first introduced by Keppler *et al*. (2006) and later confirmed by a number of studies (Brüggemann *et al*., 2009; Bruhn *et al*., 2012; Martel & Qaderi, 2017; Covey & Megonigal, 2019). The fluxes detected in our study (ranging from −28 to 37 ng g^−1^ DW h^−1^ for both species and years) were comparable to those reported by Dueck *et al*. (2007) (−10 to 42 ng g^−1^ DW h^−1^), but our better detection limit (Table 1) allowed us to confidently detect fluxes of this magnitude, especially for Scots pine in 2019. Therefore, the lack of confirmation of CH_4_ emission reported in some previous studies might have been due to their detection limits being too high.

We acknowledge that the process of CH_4_ production cannot be directly assumed to be aerobic purely because the emissions are measured from shoots (Covey & Megonigal, 2019). Our experimental setup, however, limits the CH_4_ production in potential sources other than needles to a minimum – although boreal trees, including Scots pines, have been shown to emit soil‐derived, anaerobically produced CH_4_ from their stems, substantial transport of CH_4_ from the soil is mainly associated with locations where the water table is relatively high (Machacova *et al*., 2014). Moreover, in well‐drained upland sites, shoot CH_4_ emissions exceeded stem emissions 41‐fold (Machacova *et al*., 2016). It should therefore be noted that our measured saplings were planted into well‐drained pots on a sand bed and only moderately irrigated. Although the presence of anaerobic microsites in the pots cannot be fully ruled out as possible sites of microbial methanogenesis, CH_4_ formation at such sites would be negligible relative to the quantities of CH_4_ we measured from the shoots. Therefore, it is most likely that the CH_4_ was produced in the needles.

Our results for the Norway spruce do not show evidence of CH_4_ emissions. Other authors have reported net uptake of CH_4_ by shoots of Norway spruce (Sundqvist *et al*., 2012), indicating that there may be parallel, possibly microbial, processes contributing to the overall canopy‐level CH_4_ fluxes of conifers. We observed apparent net CH_4_ uptake during individual measurements in 2019 and 2020 (Figs 1, 2, 3). These observations may indicate net CH_4_ uptake by microbial methanotrophs, which have been detected in the phyllosphere of Norway spruce (Putkinen *et al*., 2021). The concurrent uptake of CH_4_ with emissions would explain the lower CH_4_ fluxes observed in Norway spruce shoots. The observed apparent CH_4_ uptake rates, however, generally remained within the measurement uncertainty; hence, our data provides no further evidence for or against CH_4_ uptake.

### Methane emissions are controlled by solar radiation and further enhanced by temperature

The CH_4_ emissions correlated with PAR and global radiation in Scots pine (Fig. 4), making our study one of the first to demonstrate solar radiation as a driver of aerobic CH_4_ emissions from the shoots of conifers under ambient conditions. The only field study that has addressed this question before showed no correlation with solar radiation in Japanese cypress (Kamakura *et al*., 2012), although sunshine exposure had already been shown to increase CH_4_ emissions from living plants in the laboratory by Keppler *et al*. (2006).

Previously, positive correlations between CH_4_ emissions and radiation (and air temperature) have been shown in laboratory conditions with dried and fresh bulk plant biomass, structural components like pectin and lignin, and surface waxes (McLeod *et al*., 2008; Vigano *et al*., 2008; Bruhn *et al*., 2014). In these studies, UV‐light, especially in the UVB region, was identified as a key driver for CH_4_. These experiments were, however, conducted mostly at UV radiation levels (up to 7 W m^−2^ UVB) that were much higher than typical UVB radiation in Finland during springtime (daily maximum *c*. 1–4 W m^−2^ UVB in March–June). The CH_4_ emissions in our study may also depend on radiation in other spectral regions, for example UVA and blue light, which has been recently shown to induce aerobic CH_4_ production in canola (Martel & Qaderi, 2019). Besides the apparent direct effects of solar radiation, our measurements also show a correlation between CH_4_ emission and CO_2_ uptake; therefore, it is possible that the source process of the CH_4_ emissions is, to at least some extent, associated with other light‐driven tree physiological processes, such as the interaction of the methionine cycle and C_1_ metabolism, which is closely linked to photosynthesis.

In our 2019 campaign, we found a strong correlation between CH_4_ emissions and air temperature for Scots pine shoots in direct sunlight, whereas in cloudy conditions there was no correlation (Fig. 5a). Notably, although sunshine generally increases the air temperature close to the ground as well as in the closed measurement chambers early in the spring in Finland, in April–May of 2019 PAR only explained *c*. 4% of the changes in ambient air temperature. Temperature‐driven CH_4_ release from plants and plant compounds has been reported in multiple studies (Keppler *et al*., 2006; Vigano *et al*., 2008; Bruhn *et al*., 2009); however, these experiments were conducted in laboratory conditions and mostly outside the ambient temperature range (with temperatures as high as 80°C). Our results show that even on warmer days the CH_4_ emissions remained low if the sky was overcast, whereas in warmer and sunny conditions the effect of solar radiation on CH_4_ emissions was enhanced (Fig. 5b), pointing towards a temperature‐by‐light interaction effect. Thus, although air temperature is not an independent driver of shoot CH_4_ emissions in ambient spring conditions in Finland, where daily mean temperature generally remains below 20°C, even in the 5–20°C temperature range, increasing temperature gradually increases shoot CH_4_ emissions during sunny days.

We note that photosynthesis and other metabolic activities are affected by leaf temperature, which in sunlit boreal conditions can be up to 10°C higher than air temperature, and these activities are affected in the short‐term by variations in radiation input caused by clouds (Ansari & Loomis, 1959; Leuzinger & Körner, 2007; Helliker & Richter, 2008). It is thus possible that the observed relationship between light and CH_4_ emissions was partially driven by greater warming of needles during high PAR conditions. Nevertheless, our finding of very low emissions on cloudy but warm days clearly demonstrates that needle temperature may be a modulator of light‐dependent CH_4_ emissions, but not the principal driver of such processes.

### No evidence for a relationship between spring advance and methane emissions

In the campaign of 2020, when measurements continued throughout the whole spring season, we observed a gradual increase in the CH_4_ emissions from the Scots pines in late winter towards the spring. CH_4_ emissions reached their maximum *c*. 2 wk before the first visible signs of bud growth and the increase in photosynthetic activity (*F*_v_/*F*_m_) (Fig. 1). Evergreen conifers in boreal regions are exposed to photo‐oxidative stress during early spring when the increasing levels of light energy cannot yet be utilized as photosynthesis is inhibited due to low temperature (Karpinski *et al*., 1993; Ottander *et al*., 1995; Vogg *et al*., 1998). Moreover, previous studies show that the production of ROS, which is induced by a number of plant stressors, may initiate the formation of CH_4_ within the plant (Messenger *et al*., 2009b; Wang *et al*., 2009; Bruhn *et al*., 2012; Liu *et al*., 2015). Although our results do not rule out the seasonal increase in solar radiation as an explanation for the increasing trend in CH_4_ fluxes between March and April, the timing of the highest measured CH_4_ emissions of Scots pine in early to mid‐April raises the question of whether the increased susceptibility of the shoots to light before the full upregulation of the photosynthetic machinery promotes the formation of CH_4_, possibly via ROS‐related pathways (Messenger *et al*., 2009a).

After the gradual increase in early March to mid‐April (Fig. 2a), CH_4_ emissions did not continue to increase with further advancing of the spring. This was despite increases in solar radiation and in the ratio of newly grown (*Y*_0_) to year‐old (*Y*_1_) shoot biomass in the measurement chambers. Thus, we found no evidence for a difference in CH_4_ emissions from *Y*_0_ and *Y*_1_ shoots, whereas such differences were found in methanol emissions from Scots pine shoots (Aalto *et al*., 2014), indicating buds and growing shoots act as a strong source of methanol but not CH_4_.

### Uncertainties in the measurements

Our results show variability in the observed CH_4_ fluxes between the two measurement years for both Scots pine and Norway spruce, with notably larger variance in the observed fluxes of 2020 (Fig. 2). To some extent, these differences arise from technical limitations in the measurements of leaf‐level CH_4_ fluxes that are very small relative to the atmospheric background concentration (Kohl *et al*., 2021). The variation in the observed CH_4_ emission ranges of Scots pine shoots between the years can be furthermore explained by differences in the growth patterns of the saplings that resulted in a lower needle biomass : chamber volume ratio in 2020; the measured biomass values for the Scots pine shoots were significantly smaller in 2020 than in 2019 (Table 1). As a result, the CH_4_ concentration changes during the chamber closure were smaller and, consequently, the measurement uncertainty was larger due to a higher proportion of the fluxes being close to the detection limit. Similarly, the lower needle biomass : chamber volume ratio could also explain why the measurements provided generally inconclusive evidence for CH_4_ emissions from the shoots of the Norway spruces. Finally, linear models with PAR, temperature and the uptake rate of CO_2_ all predicted very similar if not identical CH_4_ emission rates for the Scots pine in both years (Figs 3, 6), validating our findings about the relationship of these factors to the CH_4_ emissions.

### Conclusions

In conclusion, our study provides evidence that conifers emit CH_4_ from their shoots, suggesting that the evergreen boreal forest canopies may decrease the CH_4_ sink strength of boreal upland forests. We identified solar radiation as a driver for shoot CH_4_ emissions through a temperature‐by‐light interaction effect. Measurements conducted under more controlled conditions will help to further clarify the individual effects of light and temperature on CH_4_ production in the needles. We also demonstrated that in the springtime, the environmental conditions in the boreal regions, potentially together with the physiological state of the trees, favour CH_4_ formation in the shoots of evergreen conifers. Although our work increases our knowledge of canopy level CH_4_ fluxes from conifers by providing the first results of springtime CH_4_ emissions, long‐term measurements are needed to characterize the seasonality of these fluxes in order to evaluate their significance for the CH_4_ exchange at ecosystem level.

## Author contributions

The study was conceptualized by M Pihlatie, SAMT, LK and MK. The measurements were conducted by SAMT, M Patama, AZ, AL, RL and LK. The data analysis was conducted by SAMT, MK and LK. M Pihlatie, AL, MK and LK provided supervision and guidance to graduate students. SAMT wrote the first draft of the manuscript, all other co‐authors contributed to the final version.

## Supporting information


**Fig. S1** Example plots showing the adjustment of the start and end times of measurement closures, based on a graphical assessment of CH_4_ and CO_2_ mixing ratios as a function of time.
**Table S1** Table presenting the chamber background CH_4_ fluxes defined from empty chamber measurements, and shoot CH_4_ fluxes defined as shoot chamber fluxes before scaling to dry weight and after subtracting the chamber background flux.
Please note: Wiley Blackwell are not responsible for the content or functionality of any Supporting Information supplied by the authors. Any queries (other than missing material) should be directed to the *New Phytologist* Central Office.

## Data Availability

The original data are available at 10.5281/zenodo.6367101. Additional data related to this article may be requested from the corresponding author (SAMT).
