# Supplementary material for: Solar radiation drives methane emissions from the shoots of Scots pine
Source: New Phytol. 2022 Apr 12;235(1):66–77. doi: 10.1111/nph.18120 (PMC9325065; doi:10.1111/nph.18120)
Supplement: Supplementary file 1 — Fig. S1 Example plots showing the adjustment of the start and end times of measurement closures, based on a graphical assessment of CH4 and CO2 mixing ratios as a function of time. Table S1 Table presenting the chamber background CH4 fluxes defined from empty chamber measurements, and shoot CH4 fluxes defined as shoot chamber fluxes before scaling to dry weight and after subtracting the chamber background flux. Please note: Wiley Blackwell are not responsible for the content or functionality of any Supporting Information supplied by the authors. Any queries (other than missing material) should be directed to the New Phytologist Central Office. [file NPH-235-66-s001.pdf]

New Phytologist

***New Phytologist* Supporting Information**

Article title: Solar radiation drives methane emissions from the shoots of Scots pine

Authors: Tenhovirta, Salla; Kohl, Lukas; Koskinen, Markku; Patama, Marjo; Lintunen, Anna; Zanetti, Alessandro; Lilja, Rauna; Pihlatie, Mari

Article acceptance date: 11 March 2022

The following Supporting Information is available for this article:

Fig. S1 Example plots showing the adjustment of the starting and ending times of measurement closures, based on the graphical inspection of CH_4 and CO_2 mixing ratios as a function of time.

Table S1 A table presenting the chamber background CH_4 fluxes defined from empty chamber measurements, and shoot CH_4 fluxes defined as shoot chamber fluxes before scaling to dry weight and after subtracting the chamber background flux.

Fig. S1 Example plots of the adjusted closure start and end times based on the graphical inspection of closures, showing the changes of the mixing ratios (p.p.m, parts per million) of CH_4 (solid black line) and CO_2 (dotted blue line) over time (sec). The 0-point for time indicates the start of the closure after adjustment. The vertical lines show the un-adjusted (dashed red line) and adjusted (solid grey line) start and end times of the closures. Plot titles include the chamber ID and measurement date and time. Note that the scales of X axis and Y axis for the CH_4 mixing ratio varies between the plots.

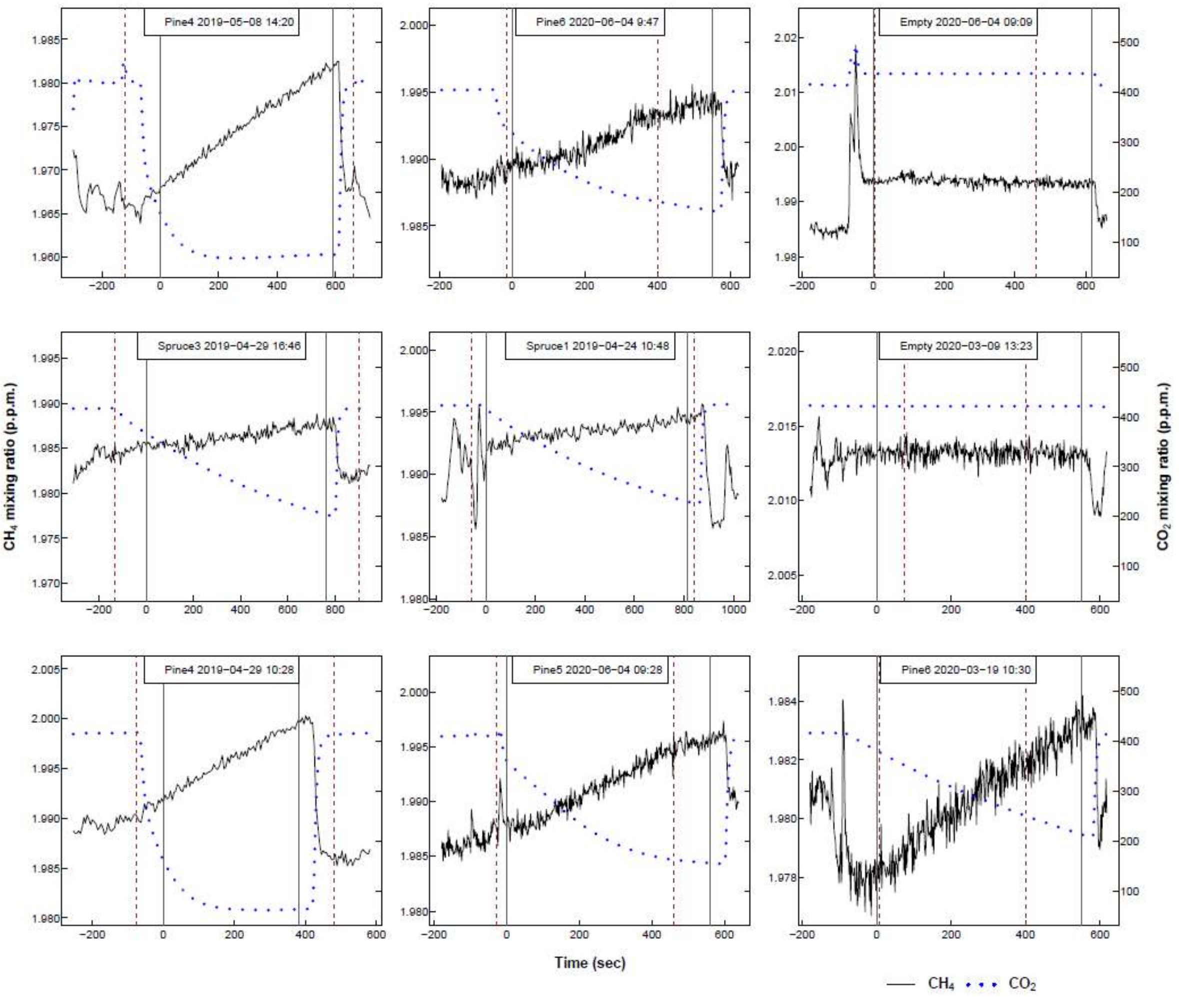

Table S1 Chamber background CH_4 fluxes defined from empty chamber measurements, and shoot CH_4 fluxes defined as shoot chamber fluxes before scaling to dry weight and after subtracting the chamber background flux.

		Year		
**Measure**	**Unit**	**2019**	**2020**	**Tree species**
Background flux[a]	ng CH_4 h^{-1}	27.38 ± 5.43	11.75 ± 6.06	Empty
Shoot flux[b]	ng CH_4 h^{-1}	112.95 ± 6.47	39.29 ± 4.47	Scots pine
		9.53 ± 4.51	10.55 ± 6.95	Norway spruce

[a] Background CH_4 emission of the empty chamber (mean ± standard error). [b] CH_4 emission of spruce or pine shoots (mean ± standard error), the background flux subtracted, and not scaled to shoot dry weight.
